# A case report of tongue metastasis from lung squamous cell carcinoma and literature review: Erratum

**DOI:** 10.1097/MD.0000000000008909

**Published:** 2017-11-27

In the article, “A case report of tongue metastasis from lung squamous cell carcinoma and literature review”,^[1]^ which appeared in Volume 96, Issue 40 of *Medicine*, Dr. Yi-ping Han appeared incorrectly as Yipin Han.
